# Time-based prospective memory in preschoolers – the role of time monitoring behavior

**DOI:** 10.3389/fpsyg.2024.1276517

**Published:** 2024-02-07

**Authors:** Elżbieta Szpakiewicz, Natalia Maja Józefacka

**Affiliations:** ^1^Institute of Psychology, Jagiellonian University, Krakow, Poland; ^2^Institute of Psychology, University of the National Education Commission, Krakow, Poland

**Keywords:** prospective memory, time-based, cognitive abilities, monitoring, children, preschool-age

## Abstract

**Background:**

Time-based prospective memory (TBPM) refers to the ability to remember to perform an intended activity at a specific time in the future or after a specific time interval. This article reviews TBPM memory in preschool children and explores the role of time monitoring behavior in TBPM performance.

**Methods:**

A total of 242 preschool-aged children (aged 2–6) performed a prospective memory task, wherein prospective memory accuracy, ongoing task performance, and time monitoring activity were assessed. Additionally, the study examined the relationship of various cognitive abilities to TBPM performance through the use of appropriate cognitive tasks.

**Results:**

The first signs of TBPM were observed in children as young as 2 years old. No significant age differences were identified; preschoolers can perform a delayed intention on their own initiative at a certain point in the future only to a minimal extent. The majority of variance in TBPM performance could be explained by time checking behavior.

**Conclusion:**

The current study indicated that even 2-year-olds can perform TBPM at a basic level when the task is sufficiently understandable. While many cognitive abilities are correlated with TBPM performance, it appears that only time checking behavior plays a significant role in TBPM among preschoolers.

## 1 Introduction

Every time a child has to remember to take their beloved teddy bear on vacation, send a message to a parent or brush their teeth before going to bed, they need to remember not only what they are supposed to do (i.e., retrospective memory; RM), but also remember to execute this intended action at the appropriate moment in the future (i.e., prospective memory; PM). Einstein and McDaniel (1990) suggested a distinction between event-based prospective memory (EBPM) and time-based prospective memory (TBPM). While EBPM refers to the ability to remember to perform an intended activity when the appropriate moment to initiate the delayed intention is indicated by a predetermined event (i.e., come to a special snack when requested by the parent), TBPM is about remembering to perform some action at a certain point in the future or after a certain time interval (i.e., come to a special snack in 10 min).

A PM ability is crucial as it plays a significant role in daily life activities, independence, and academic success (e.g., Meacham, 1982; Kvavilashvili et al., 2001; Mahy et al., 2014). Therefore, several authors have argued that PM may begin to manifest itself very early in development (Meacham and Colombo, 1980; Winograd, 1988). Actually, available evidence suggests that children as young as 2 years old are able to succeed in the task of EBPM (Somerville et al., 1983; Ślusarczyk et al., 2018), and their EBPM becomes more efficient as they age (see Mahy, 2022, for a review). There are only a few studies that have investigated the mechanisms and processes that lead to successful TBPM performance in preschool and school children (e.g., Ceci and Bronfenbrenner, 1985; Kerns, 2000; Mackinlay et al., 2009; Aberle and Kliegel, 2010). The findings of this research suggest that TBPM tasks can be performed by school-aged children. However, preschoolers demonstrated significant difficulties in these tasks. It is worth noting that the youngest children ever included in research on TBPM were 4 years old (Geurten et al., 2016). Nonetheless, it remains unclear whether younger preschoolers are able to remember to perform a delayed intention on their own initiative at a certain point in the future. The present study explores this question for the first time.

The most significant issue in the research on TBPM in children is their lack of familiarity with clocks. Aberle and Kliegel (2010) employed an intriguing procedure to circumvent this obstacle by using an hourglass. Given that the PM abilities of very young children may be best revealed in tasks and situations that are familiar and well understood by them (*cf.* to event-based tasks, e.g., Somerville et al., 1983; Ślusarczyk et al., 2018), in research with the youngest preschoolers (i.e., 4-year-olds and younger), this modification seems to be insufficient, and an additional motivating factor should be applied. In the current study, we decided to modify the procedure developed by Aberle and Kliegel (2010) by incorporating the context of engaging in play with a plush mascot.

From a review of research on EBPM in very young children (e.g., Somerville et al., 1983; Kliegel and Jäger, 2007; Ślusarczyk et al., 2018), it can be assumed that preschoolers of TBPM can occur in children under the age of 4. However, regarding the two frameworks of PM: the multi-process model (McDaniel and Einstein, 2000) and the Executive Framework (Mahy et al., 2014), one can argue that TBPM tasks are more resource demanding than EBPM tasks due to the need to actively and strategically monitor the passage of time (see, e.g., Costermans and Desmette, 1999). Hence, it appears that preschoolers will demonstrate a capacity to perform TBPM tasks only to a minimal extent (as compared to their performance on EBPM tasks).

The lack of accuracy in TBPM performance in young children seems to be related to a deficient strategic use of time monitoring (i.e., monitoring if there is exact time to perform the PM task) or a lack of time checking behavior. However, future research is needed to explore these hypotheses. Due to the limited research on TBPM in preschoolers, valuable insights can be drawn from studies conducted with adults and elderly individuals. Research with adults indicated positive correlations between the number of clock checks and PM task accuracy (Mioni and Stablum, 2014; Mioni et al., 2020). Moreover, Schmidt et al. (2023) found a decrease in TBPM with advancing age that was attributed to reduced clock checking within 30 s before intention completion. Similarly, in research with schoolchildren, more frequent time checking was found to be related to better TBPM performance (Voigt et al., 2011). Additionally, Ceci and Bronfenbrenner (1985) found that older children showed more strategic clock checks than younger children and were more successful in the TBPM task. Therefore, the poor TBPM task performance appears to result from either insufficient monitoring or ineffective monitoring strategies.

Previous research indicated that the most efficient strategy for timely accomplishment of the intended task is to check the clock more frequently as the target time is approaching (e.g., Mioni and Stablum, 2014; Vanneste et al., 2016; Mioni et al., 2020). Therefore, in the current study, we decided to divide the time interval between forming an intention and its retrieval into two periods of 15 s each, to examine the strategy used by children (see Vanneste et al., 2016, for a similar procedure in a study with young and older adults).

Since TBPM is presumed to rely specifically on self-initiated intellectual activity and detecting time-based task cues requires cognitive resources as individuals need to strategically monitor the appearance of the PM target-time, it has been suggested that problems with executive functions (EF) or other cognitive abilities may contribute to failures in TBPM performance. The Executive Framework (Mahy et al., 2014) argues that EF drive PM development in children, and age-related improvements in PM can be predicted by the development of specific EF. Although many studies agree on the existence of a link between PM and other cognitive abilities, so far there is no consensus on how they are specifically associated with PM (see Zuber et al., 2019, for an overview). A possible mechanism in TBPM development might be seen, for example, in the development of working memory (WM) (Kerns, 2000; Mackinlay et al., 2009; Aberle and Kliegel, 2010; Kretschmer et al., 2014; Voigt et al., 2014) or inhibition (Kerns, 2000; Mäntylä et al., 2007). Moreover, in the adult studies, fluid intelligence and psychomotor speed have also been discussed as essential mechanisms that may facilitate PM functioning (Salthouse et al., 2004; Smith and Bayen, 2005).

Time perception, encompassing the ability to assess the duration of time or comprehend its passage (Khan and Dixit, 2006), could impact a child’s performance in the TBPM tasks, as they require monitoring elapsed time. Therefore, developing skills in time perception might affect the ability to perform the TBPM task. Several researchers have suggested a connection between time perception, time estimation, and TBPM accuracy (e.g., Graf and Grondin, 2006; Block and Zakay, 2008). Similarly, time perception was found to predict PM accuracy in children (Mioni et al., 2017). However, Mackinlay et al. (2009) reported that time estimation could not explain any substantial proportion of age-related variance in TBPM performance in schoolchildren. Therefore, future research is required to investigate the significance of time checking behavior in PM.

### 1.1 Current study

Although there are few TBPM studies involving preschoolers, the question about the earliest age at which TBPM skills can be observed is still an unresolved issue. Therefore, the first goal of the current study was to find the first signs of TBPM. To the best of our knowledge, our sample consisted of the youngest children that have ever participated in research on TBPM. By extrapolating from previous studies involving very young preschoolers (e.g., Somerville et al., 1983; Ślusarczyk et al., 2018) and based on theoretical propositions that suggest that PM is functionally significant and may manifest itself very early in development (Meacham and Colombo, 1980; Winograd, 1988), we expected that 2-year-olds would be able to perform the TBPM task, at least at a basic level (1st hypothesis).

As a second research question, we aimed to investigate potential age differences among preschoolers in the domain of TBPM. Given the intensive development of various general cognitive abilities in preschool-aged children (e.g., Zelazo et al., 2003 for EF; He et al., 2019 for social WM), along with the notable enhancements in EBPM, especially observed between ages 3 and 6 (e.g., Guajardo and Best, 2000; Kliegel and Jäger, 2007; Wang et al., 2008), we anticipated observing age-related variations in TBPM task performance. It is essential to note, however, that our study is exploratory in nature. Thus far, TBPM has not been systematically investigated within a cohort of preschool-aged children spanning the entire age range. Assuming TBPM falls within the cognitive abilities domain and recognizing the most significant developmental leap in cognitive development occurs between ages 3 and 5, we expect substantial differences in TBPM task performance, specifically between children aged 2 and 3 and those in older age groups (5–6 years old) (2nd hypothesis).

TBPM is assumed to be dependent on self-initiated cognitive activities, such as time monitoring (d’Ydewalle et al., 2001). In preschoolers, monitoring ability is still in the early stages of development. This developmental stage can lead to challenges in performing TBPM tasks. Therefore, as a third research aim, we investigated the role of time monitoring in preschoolers’ TBPM. We hypothesized that preschoolers who engage in more frequent time checking behavior would exhibit improved TBPM performance (3rd hypothesis).

Considering that in preschoolers, many cognitive abilities are closely associated but distinct constructs [*cf.*, (Ger and Roebers, 2023) for EF, WM, and intelligence], this study also aimed to explore potential cognitive correlates of TBPM in preschoolers. Therefore, we examined the relationship between TBPM and fundamental cognitive abilities, such as WM, inhibitory control, fluid intelligence, selective attention, time perception, switching, RM, and language abilities. This investigation could provide correlational evidence for the possible association between cognitive resources and TBPM in preschoolers. Furthermore, our final research question focused on determining whether any of these cognitive abilities would emerge as significant predictors of preschoolers’ TBPM (4th hypothesis).

In summary, the current research was conducted to provide an overview of TBPM in preschoolers and to explore factors influencing its development.

## 2 Materials and methods

### 2.1 Participants

Two hundred and fifty-five children aged 2 to 6 years (28–82 months) participated in the study. Children were divided into five age groups: 2-, 3-, 4-, 5-, and 6-year-olds. Fifteen children who could not report the PM instruction correctly, even after the most specific prompt (see the *Procedure* section), were excluded from the final sample and analyses (four 2-year-olds, six 3-year-olds, three 4-year-olds, and two 6-year-olds). Therefore, the final sample consisted of 240 children (*M_age_* = 54.19 months, *SD* = 15.91), 133 of which were girls (55%; there was no significant difference in age between the two gender groups). None of the participants reported a history of neuropsychopathology or psychopathology (assessed by their preschool teachers who had received prior instructions) and had intellectual deficits. Children were recruited through their preschools; informed written parental consent was obtained for all participants. The recruitment involved 11 different preschools, and parents were informed about the research through communication by teachers. It’s important to note that participation was entirely voluntary, and children had the option to choose not to take part if they did not wish to. All children came from upper-working or lower-middle-class families and lived in southern Poland, predominantly (80%) in the urban areas. Table 1 displays number of children, gender distribution, and mean age per age group of the final sample.

**Table 1:** Number of participants, gender distribution and mean age per age group of the final sample.

	*N*	*Girls*	*Age range*	*M*_*age*_ *(SD)*
Overall	240	133	28–82	54.19 (15.91)
2-years	47	26	28–35	32.17 (1.79)
3-years	47	29	37–47	42.09 (3.79)
4-years	47	25	48–59	54.11 (3.72)
5-years	50	27	60–71	65.60 (3.57)
6-years	49	26	72–82	75.37 (2.42)

*N*, number of participants; *Age*, age in months.

### 2.2 Design

The design was an independent measures design, with Age (2-, 3-, 4-, 5- vs. 6-years) as a between-subjects factor.

### 2.3 Measures

#### 2.3.1 Prospective memory task

For the PM task, a modified version of the procedure described by Aberle and Kliegel (2010) was applied. Children were asked to play a standard version of the Memory/Pairs game with the experimenter for about 5 min (*the ongoing task; OT*) and to turn the hourglass whenever the sand had run to the bottom bulb (*the prospective memory component*). As one time circle of the hourglass lasted 30 s, the maximal number of turns was 10 times. To make the task interesting enough for very young children, the child was introduced to the owl mascot named Clara (see the *Procedure* section). The OT performance was calculated by summing up the number of pairs of Memory/Pairs cards the children found. Participants played the game independently, and a total of 30 matches were possible. As a measure of PM performance, one point was given for each turn of the hourglass, and the total score was the number of hourglass turns, with a maximum score of 10. Moreover, the number of times the child glanced at the hourglass and the moment they did so were recorded as a measure of monitoring accuracy. We decided to partition the delay period, which lasted 30 s in each instance, into 2 intervals of 15 s to explore when exactly the child checks the passage of time. For all measurements, Kendall’s *W* = 1.00, indicating complete agreement between competent raters.

#### 2.3.2 Time perception

Time perception was calculated as the mean value of the percentage of errors made in the task *Time reproduction* at 4, 6, 8, and 10 s, as well as the percentage of errors in *Time differentiation*. A negative interpretation is expected (i.e., a higher time perception index indicates poorer time perception in children). Prior to the analysis, Z-scores were applied to standardize the scores.

##### 2.3.2.1 Time reproduction

The child was to ring the bell for as long as the experimenter had rung before. A practice trial (ringing for 2 s) was carried out to ensure that the child understood the instruction. Then 4 trials were conducted: 4, 6, 8 and 10 s. The absolute difference between the time the child rang the bell and the target number of seconds was calculated for each trial. The percentage of child’s error in a given trial was then calculated. Inter-rater reliability was high for both practice and test trials, with Kendall’s *W* ranging from 0.96–0.99.

##### 2.3.2.2 Time differentiation

The child was shown two bells: black and gold. The experimenter rang one bell and then another, and the child’s task was to judge which one rang longer. Three trials were administered: 1. the black bell rang for 4 s and the gold one for 6 s; 2. black for 9 s and gold for 6 s; 3. black for 3 s and gold 2 s. The child received 1 point for pointing to the correct bell (range: 0–3). Coding was reliable, Kendall’s *W* = 1.00.

#### 2.3.3 Retrospective memory: Auditory memory task

The sub-test Auditory memory task from the IDS-P Intelligence and Development Scales for Pre-school Children (Grob et al., 2015) Polish-version was applied. The child was asked to recall a story heard 30 min earlier. When the child failed to recall the story spontaneously, additional detail questions were asked. Each spontaneous recall of the key story detail was scored 2 points and the recall after the auxiliary question – 1 point (range: 0–20).

#### 2.3.4 Working memory

WM was calculated as the mean value of the percentage of correct answers in the *Verbal Working Memory* and *Nonverbal Working Memory.*

##### 2.3.4.1 Verbal working memory: the Forward and Backward Digit Span Tasks

To measure verbal WM, children completed the digits forward and backward subscales from the Wechsler Intelligence Scale for Children – Revised (Matczak et al., 2008). In the Forward Digit Span Task, children were asked to repeat a series of numbers in the same order after the experimenter read them aloud. In the Backward Digit Span Task, children were asked to repeat a series of numbers in backward order. They began with two numbers, and after completing two trials successfully an additional number was added. The task ended when children failed on two consecutive trials. The Forward Digit Span Task was calculated by summing up the number of forward digit strings children were able to repeat accurately (max 14 points). Similarly, the Backward Digit Span Task was calculated by summing up the number of backward digit strings children were able to repeat accurately (max 14 points).

##### 2.3.4.2 Nonverbal working memory: the Forward and Backward Corsi Block-Tapping Tasks

To measure nonverbal WM, a modified child-friendly computer version of Corsi Block-Tapping Task (Corsi, 1972) was applied. The task involves a sequence of illuminated blocks (depicting, e.g., a flower, a sun, etc.) of increasing span length (from two to six pictures) to be tapped by a child in forward or backward manner. Two trials per length were given. Starting from sequences of two blocks, if the participants correctly reproduced at least one sequence of the same length, they proceeded to sequences that were one block longer. The task ended when the child failed two consecutive trials. The backward task was administered after completing the forward task, that makes the procedure comparable with the Forward and Backward Digit Span Tasks. Referring to the standard scoring procedure proposed by Corsi, the rating (0 or 1) for each trial was multiplied by the number of blocks that should have been pressed. Thus, the score of Nonverbal Working Memory was ranged 0–80 (i.e., 0–40 for the Forward and 0–40 for the Backward Corsi Block-Tapping Task).

#### 2.3.5 Inhibition

The measure of inhibition was calculated as the mean value of the percentage of correct answers in the *Visual Simon Task*, the *Bear/Dragon task* and the *Day/night task*.

##### 2.3.5.1 Visual Simon Task

The Visual Simon Task is a modified version of Spatial Conflict (Gerardi-Caulton, 2000). Children were presented with two kinds of visual stimuli (yellow or blue fish) on the computer screen. Children were told to respond to stimuli by making a rightward response to a yellow fish and a leftward response to a blue fish (by pressing the appropriate button on the keyboard: yellow or blue). These stimuli were presented one at a time on either the right or left side of the screen. The location of the display on which the stimuli appear influences children’s patterns of responding by either matching (i.e., congruent trials) or not matching (i.e., incongruent trials) the side (left or right) of the correct button press associated with the color of fish. The score ranges from 0 to 13 (only for incongruent trials).

##### 2.3.5.2 Day/night task

The Day/night Stroop-like task (Gerstadt et al., 1994; review: Montgomery and Koeltzow, 2010) requires that children say the opposite of what the stimulus cards represent. A total of 20 cards (4 trial cards and 16 test cards: 8 sun, 8 moon) were used for testing. On the front of half the cards was the picture of a large bright-yellow sun on a white background. On the front of the other cards was a picture of a yellow moon and silver star against a black background. Introduction to the task began with the experimenter presenting the white, sun card, instructing the child to say “Night” when shown that card. Next the experimenter showed the child the black, moon card and instructed the child to say “Day” whenever shown that card. When a child was correct on two consecutive practice trials, the child proceeded to testing on the standard condition. The cards were presented in the pseudorandom order of suns and moons. The accuracy (the number of correct items out of 16) is recorded. Inter-rater reliability was high for both practice and test trials, Kendall’s *W* = 1.0.

##### 2.3.5.3 Bear/Dragon task

The Bear/Dragon task (Reed et al., 1984) assesses the ability to inhibit or activate a motor response following a rule, in a similar way as in a *go no-go* task (or Simon Says task). The experimenter introduces children to a “nice” bear puppet and a “naughty” dragon puppet. The children are told that in this game, they are to do what the bear asks them to do (e.g., “touch your nose”), but not to do what the dragon asks. After practicing, there are 10 test trials with the bear and dragon commands in a semi-alternating order. The score ranges from 0 to 5 (only for *no-go* responses); Inter-rater reliability was high for both practice and test trials, Kendall’s *W* = 1.0.

#### 2.3.6 Switching: Children Card Sort

The Children Card Sort (Jabłoński et al., 2018) is a method based on the Dimensional Change Card Sorting, which was originally published by Zelazo (2006). The Children Card Sort has been standardized and normalized for Polish-speaking children, and it is a sample of the task-switching paradigm used for assessing cognitive flexibility in children. In the first two stages, children are required to sort a series of bivalent test cards (a house/a cat; red/blue), first according to one dimension (i.e., color), and then according to the other (i.e., shape). In the next stage, there was a new sorting rule related to the appearance of a frame on the cards: a card with a frame means sorting by color, and a card without a frame means sorting by shape. The researcher showed the child subsequent cards, recalling the sorting rule (color vs. shape) for each of them. Thus, the child had to switch from sorting cards one way to sorting them a different way. Only those children who correctly sorted at least five cards in the *color phase* and in the *shape phase* were scored in the *border phase* (see Jabłoński et al., 2012, for the same procedure). In the current study, 41 children did not meet this criterion (18 two-year-olds, 12 three-year-olds, 9 four-year-olds, and 2 six-year-olds). The result was the sum of correctly sorted cards in the *border phase* (range: 0–12).

#### 2.3.7 Planning: Tower of London

A computerized version of the Tower of London was applied (Krikorian et al., 1994). Each time, the target state was presented in the upper field of the screen. In order to match the goal configuration, the participants operated on the initial state in the lower half of the screen, using a computer-mouse to move the balls, either alone or with the help of an experimenter (if they had problems using the computer-mouse correctly). Children were told to transform the initial state into the goal state in a predetermined minimum number of moves while following five rules: (1) only one ball may be moved at a time, (2) more than one ball could not be picked up at any time, (3) the balls may not be placed anywhere else other than the three pegs, (4) a ball in the lower row cannot be moved when another ball is laying above, and (5) three balls could be placed on the left peg, two on the middle peg, and only one ball on the right peg. The task contains 1 practice problem and 12 test problems (each child had to face all the trials). The scoring method was adapted from Shallice (1982), and it involved summing the correctly solved trials within the allowed number of moves (range: 0-12).

#### 2.3.8 Selective attention

The sub-test Selective attention from the IDS-P Intelligence and Development Scales for Pre-school Children (Grob et al., 2015) Polish-version was applied. The children were asked to sort the cards depicting ducks according to the color of their beaks (yellow vs. white). Some of the cards had a yellow sun drawn on them, but the children were to ignore them. The score is the sum of correctly sorted cards within 90 s (range: 0–72).

#### 2.3.9 General intelligence: Raven’s Colored Progressive Matrices

The RCPM (Szustrowa and Jaworowska, 2003) was administered individually, without time limit, in the book format, according to Raven’s procedure (Raven et al., 1995). Children were asked to choose the missing element from six options in a drawing. One point was given for each correct answer, and the total score was the sum of the correct answers, with a maximum score of 36.

#### 2.3.10 Language ability: Picture Vocabulary Test – Comprehension

The Picture Vocabulary Test – Comprehension (Haman et al., 2012) is a normalized diagnostic tool designed to assess the language ability of Polish-speaking children aged 2–12 years. The task measured the comprehension of nouns, verbs, and adjectives. Each item was accompanied by four colored pictures. One picture depicted the target word and the three other pictures were foils. The Picture Vocabulary Test was calculated by summing up the number of correct answers (range: 0–86).

### 2.4 Procedure

The children were individually tested in a small room during two sessions separated by several days. All testing sessions were carried out in a quiet environment to ensure minimal distraction for the children. Each session, lasting approximately 40 min, was recorded. Specific tests were additionally assessed for reliability by two coders on a randomly selected 10% of the sample for each test.

At the first session, to familiarize the children with the experimenter, each child played a warm-up game with the experimenter for a couple of minutes. Then, sequentially, the tests measuring language ability, inhibition, selective attention, RM, and time perception were applied. The second session began with the PM task. After explaining the Memory/Pairs game to each child (i.e., finding as many pairs as possible in a couple of minutes), the experimenter provided the PM instruction. Each child was introduced to an owl mascot named Clara and was informed that she loves to watch the sand flow. Therefore, the child had to remember to monitor the hourglass and turn it every now and then so that time (the sand) kept flowing. Specifically, the child was told that as soon as the sand reached the bottom bulb, they had to momentarily interrupt the game, turn toward the hourglass (which was placed behind the child, out of their sight), and then turn the hourglass. The experimenter was sitting directly across from the child. To ensure appropriate comprehension and memorization of the instructions, the child was asked to repeat the PM task instructions in their own words. Whenever there was any misunderstanding, the task instructions were repeated. There were no children who were unable to repeat the instructions correctly after at most one correction. When there were no questions or misunderstandings, the child was encouraged to start playing the Memory/Pairs game. If the child forgot to turn the hourglass, after 5 s, the experimenter (without commenting on it but making sure the child noticed) turned the glass so that the sand started again and all children were presented with comparable amount of target times (see Aberle and Kliegel, 2010, for the same procedure). To ensure that children’s errors truly pertain to PM (i.e., forgetting to carry out the intention at the correct time), in cases where the child did not spontaneously perform the PM task on any of the trails, they were asked specific questions about the task they were supposed to perform. Initially, the experimenter provided a general prompt: “Was there something you were supposed to remember?” If the child did not respond, this general prompt was followed by a more specific one: “We were playing a game. Do you remember what else you had to do?” If the child still did not answer, a more detailed prompt was given: “Do you remember if there was something you were supposed to do related to Clara the owl?” If the child still did not respond, they received the most specific prompt: “Do you remember if there was something you were supposed to do when the sand fell to the bottom bulb?.” After the TBPM task, children sequentially performed tests measuring planning, WM, general intelligence, and switching.

### 2.5 Statistical analysis

An alpha level of *α* = 0.05 was established for all statistical tests as the significance cutoff. To examine gender differences in various PM measures, an independent samples *t*-test was employed, and if necessary, variance homogeneity correction was applied. To assess whether two-year-old’s performed the TBPM task, a one-sample *t*-test was utilized with a fixed reference value of zero (representing non-performance of the task). To investigate differences in performance of the TBPM task and OT between age groups and whether participants checked an hourglass, a two-way ANOVA from Ordinary Least Square (OLS) was conducted, preceded by a square root transformation to account for inconsistent variances. Post-hoc tests were conducted using a *t*-test with Bonferroni correction. Correlation matrices were built using Pearson’s correlation coefficient. Multiple regression was conducted to identify the predictors of TBPM. Before commencing the analysis, outliers in the data were excluded using the 1.5IQR method where necessary (Tukey, 1977). All calculations and graphs presented in this article were performed using Python version 3.10.7 and the following libraries: scikit-learn, scipy, pandas, numpy, seaborn, and plotly.

## 3 Results

Performance of TBPM task did not differ on any of the measures by participants’ gender (*p* of all *t*-tests >0.05, see Table 2 for details), thus we did not include gender as a variable in subsequent analyses.

**Table 2:** Gender differences in measures ( $N_{\mathrm{male}}=107$ , $N_{\mathrm{female}}=133$ ).

Measure	t	df	p	$M_{\mathrm{diff}}$	*SE_diff_*	*Min.*	*Max.*
TBPM task performance	−0.89	238	0.375	−0.27	0.30	0	10
OT performance	0.03	238	0.975	0.03	0.85	0	30
Number of prompts	1.75	238	0.082	0.37	0.21	0	4
Number of glances at the hourglass	−0.27	238	0.786	−0.15	0.55	0	22
Number of glances <15 s	0.67	238	0.504	0.15	0.22	0	10
Number of glances >15 s	−0.72	238	0.471	−0.30	0.41	0	18

Descriptive statistics were calculated for variables included in the analysis (see Table 3).

**Table 3:** Descriptive statistics of the variables in the study.

	*M*	*SD*	*Skew.*	*Kurt.*	*Min.*	*Max.*
TBPM	1.77	2.31	1.51	1.74	0	10.00
Time perception	31.99	18.94	0.52	−0.03	0	100.00
Retrospective memory	10.66	4.80	−0.10	−1.14	1	20.00
Working memory	13.28	10.38	0.79	0.20	0	49.38
Inhibition	61.90	23.95	−0.78	−0.18	0	100.00
Switching	7.38	2.20	0.62	0.03	1	12.00
Planning	3.64	2.64	0.23	−1.09	0	9.00
Attention	35.36	17.13	0.13	−0.79	2	72.00
Intelligence	16.93	6.40	0.39	−0.65	5	35.00
Language abilities	48.53	23.32	−0.38	−1.31	2	82.00
Age (in months)	54.31	16.20	−0.06	−1.40	28	82.00

### 3.1 The first signs of TBPM

**Figure 1:**
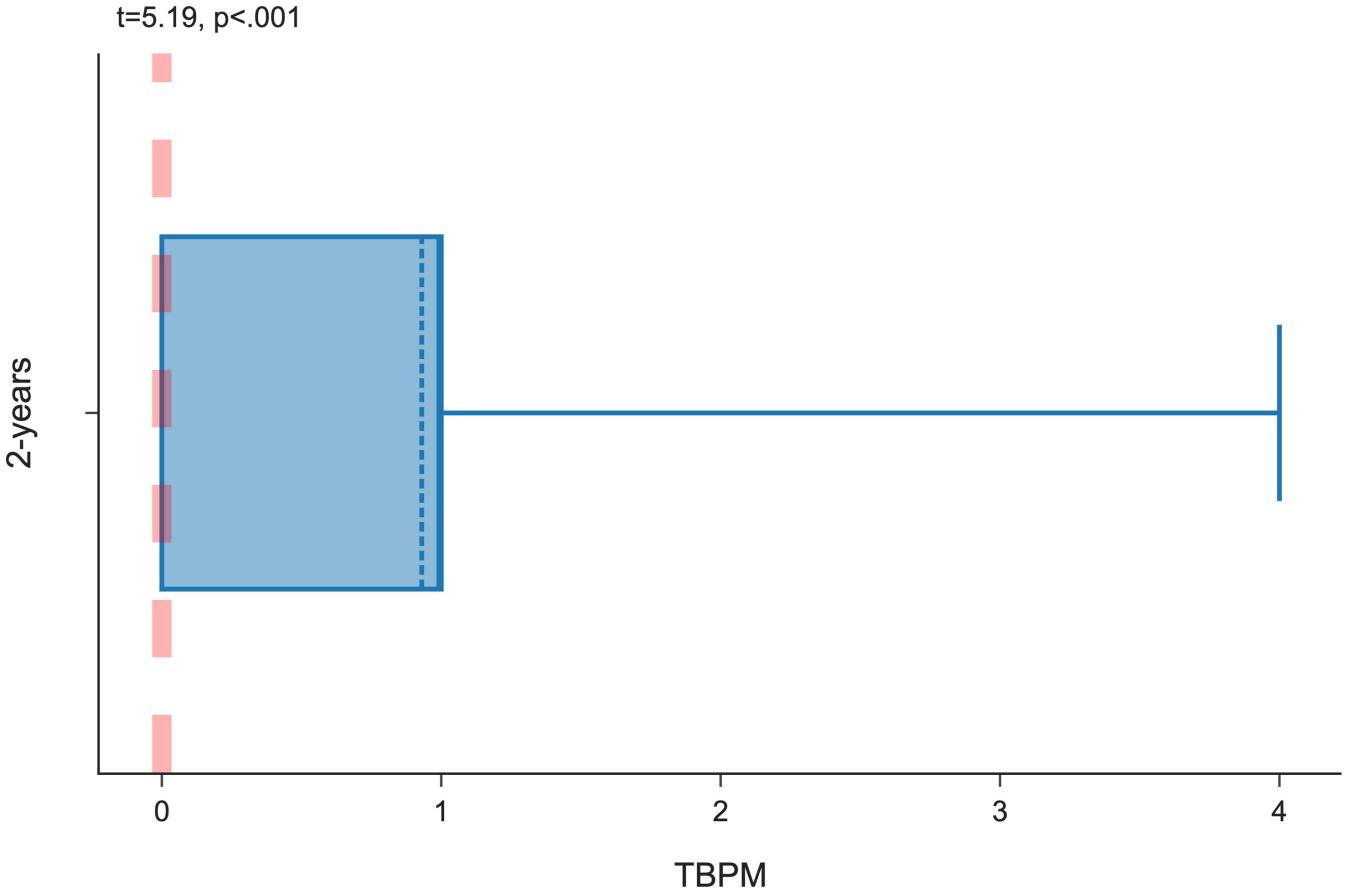
Box plot showing the performance of the TBPM task in the group of two-year-old’s ( N = 47).

To assess whether two-year-old’s perform the TBPM task, a one-sample *t*-test was conducted (see Figure 1). The reference value for the null hypothesis was set at 0, representing non-performance of the task on the TBPM scale. The analysis of the one-sample *t*-test revealed that the mean scores of two-years-old’s in the TBPM task ( M = 1.15, SD = 1.50) significantly differed from zero ( $t_{\left(46\right)}$ = 5.19, p < 0.001, Cohen’s *d* = 0.74).

In the group of two-year-old’s, 53.20% of the participants successfully completed the TBPM task with a score greater than 0, and the highest score achieved was 6 points. This task was also completed by 55.55% of three-year-old’s, 63.80% of four-year-old’s, 60.00% of five-year-old’s, and 53.10% of participants in the six-year-old group. Only 2 children scored the maximum number of points in the task (i.e., they remembered to turn the hourglass over each time): one four-year-old child and one 5-year-old child. Detailed frequency statistics of the TBPM task are presented in Table 1A (see Appendix).

### 3.2 TBPM task performance and the role of time monitoring behavior

We assumed that the task of seeking pairs within the Memory/Pairs game is equally challenging, engaging, and intriguing for all children. Therefore, there is no rationale for assessing the level of its performance. Nevertheless, we did monitor the count of identified pairs; however, this cannot be regarded as a measure of proficiency of OT. Preventively, we examined the correlation between the number of identified pairs and the level of TBPM task performance, which ultimately proved to be statistically insignificant ( r = 0.07, p >0.05).

**Figure 2:**
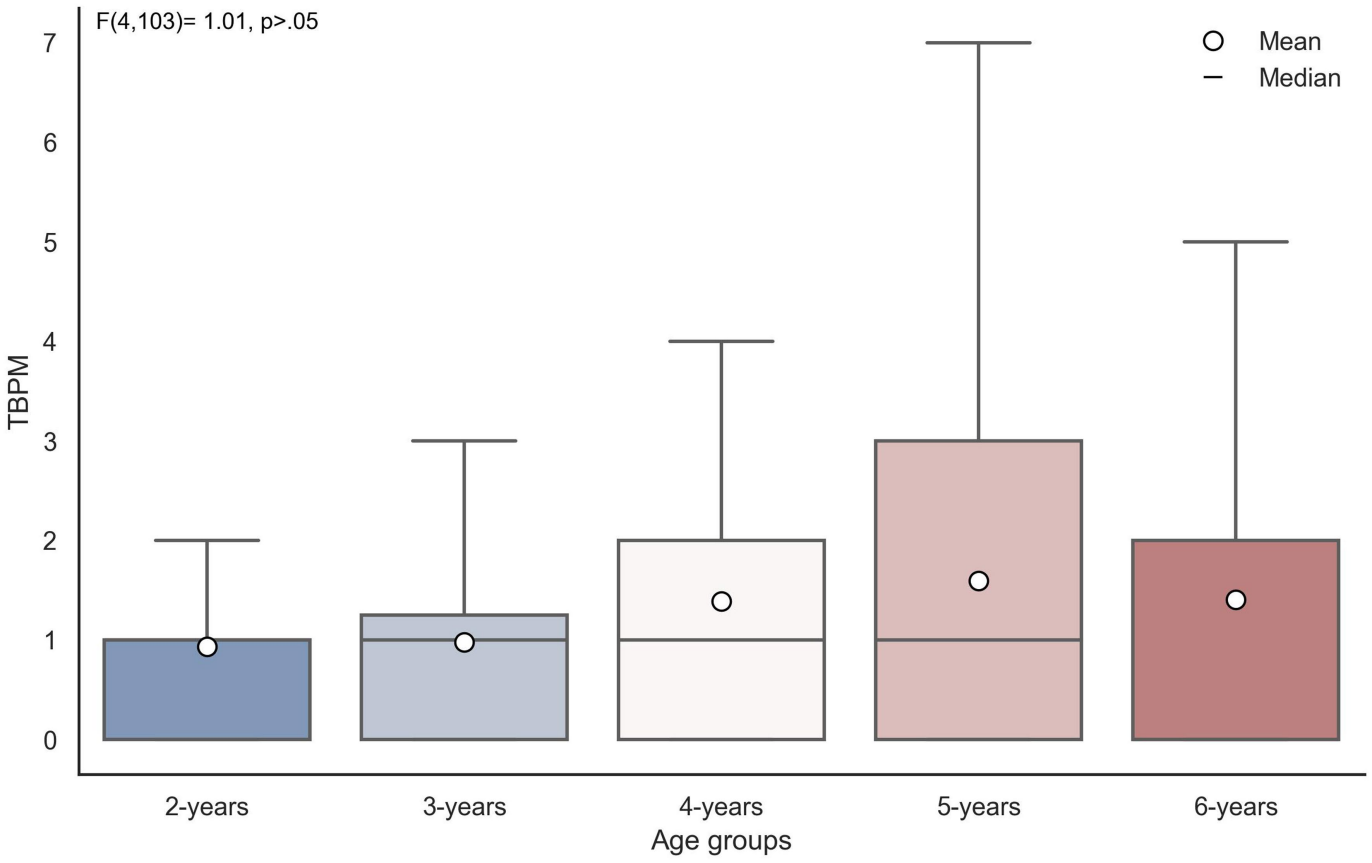
Boxplot showing no differences in TBPM between age groups.

Differences in TBPM task performance between age groups and whether the child glanced at the hourglass were examined using two-way analysis of variance (see Table 4). The results indicate that among the main effects, statistically significant differences are observed only in the scores of the TBPM based on whether the child glanced at the hourglass or not ( $F_{\left(1\right)}$ = 62.68, p < 0.001, *η^2^* = 0.27). The effect of age ( $F_{\left(4\right)}$ = 1.01, p > 0.05, *η^2^* = 0.09; see Figure 2) and the interaction between the main effects ( $F_{\left(4\right)}$ = 1.03, p > 0.05, *η^2^* = 0.11) are not statistically significant. Children who glanced at the hourglass achieved significantly higher mean scores in the TBPM task ( M = 1.63, SD = 1.71) compared to children who did not glance at the hourglass ( M = 0.17, SD = 0.64). A moderate, positive correlation was also observed between the number of glances at the hourglass and TBPM task performance ( r = 0.54, p < 0.001).

**Table 4:** Two-way ANOVA table for TBPM: age groups and number of glances at the hourglass differences.

TBPM	SS	df	F	p	$\eta^{2}$
Number of glances at the hourglass	31.66	1	62.68	<0.001	0.27
Age group	2.05	4	1.01	0.402	0.09
Number of glances at the hourglass: Age group	2.09	4	1.03	0.392	0.11

**Figure 3:**
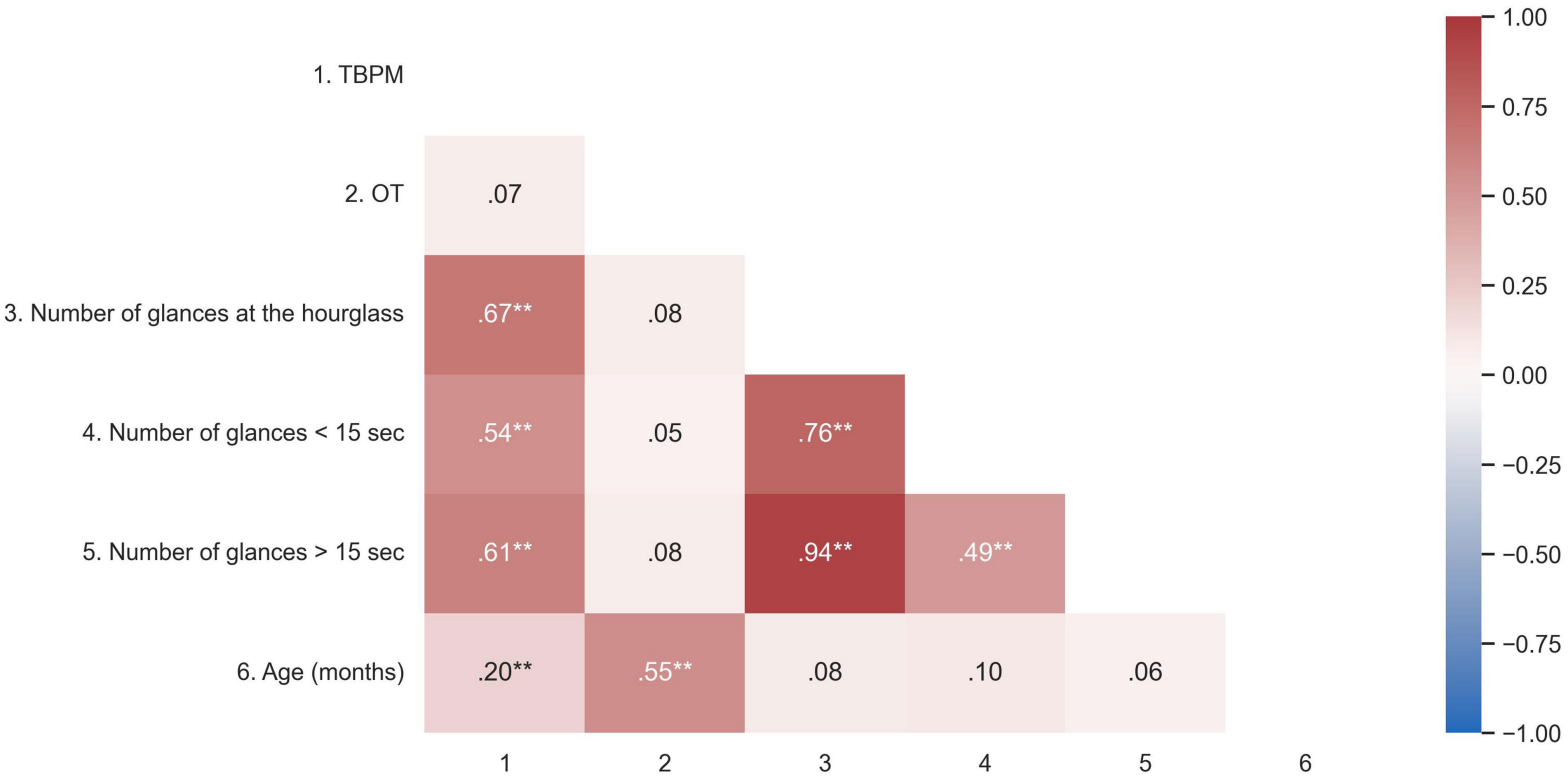
Pearson’s correlation heatmap with all PM measures and age. * p <0.05; ** p <0.01.

However, when analyzing the correlation matrix for the entire sample, positive correlations were observed between TBPM and the number of glances at the hourglass ( p < 0.01), the number of glances within 15 s ( p < 0.01), the number of glances after 15 s ( p < 0.01), and age ( p < 0.01). The mutual correlations of the remaining PM measures are presented in Figure 3. No differences in TBPM task performance in the context of specific time when the child glanced at the hourglass (first 15 s vs. last 15 s of time period) was found; *t* = 0.81, p > 0.05, Cohen’s *d* = 0.37.

Moreover, we conducted an additional analysis for groups between which we anticipated differences, combining 2-3-year-olds together and 5-6-year-olds together. These analyses yielded statistically insignificant results (*t* = 1.64; *p* = 0.103), emphasizing the lack of age differences.

### 3.3 Cognitive abilities, time perception, and TBPM task performance

**Figure 4:**
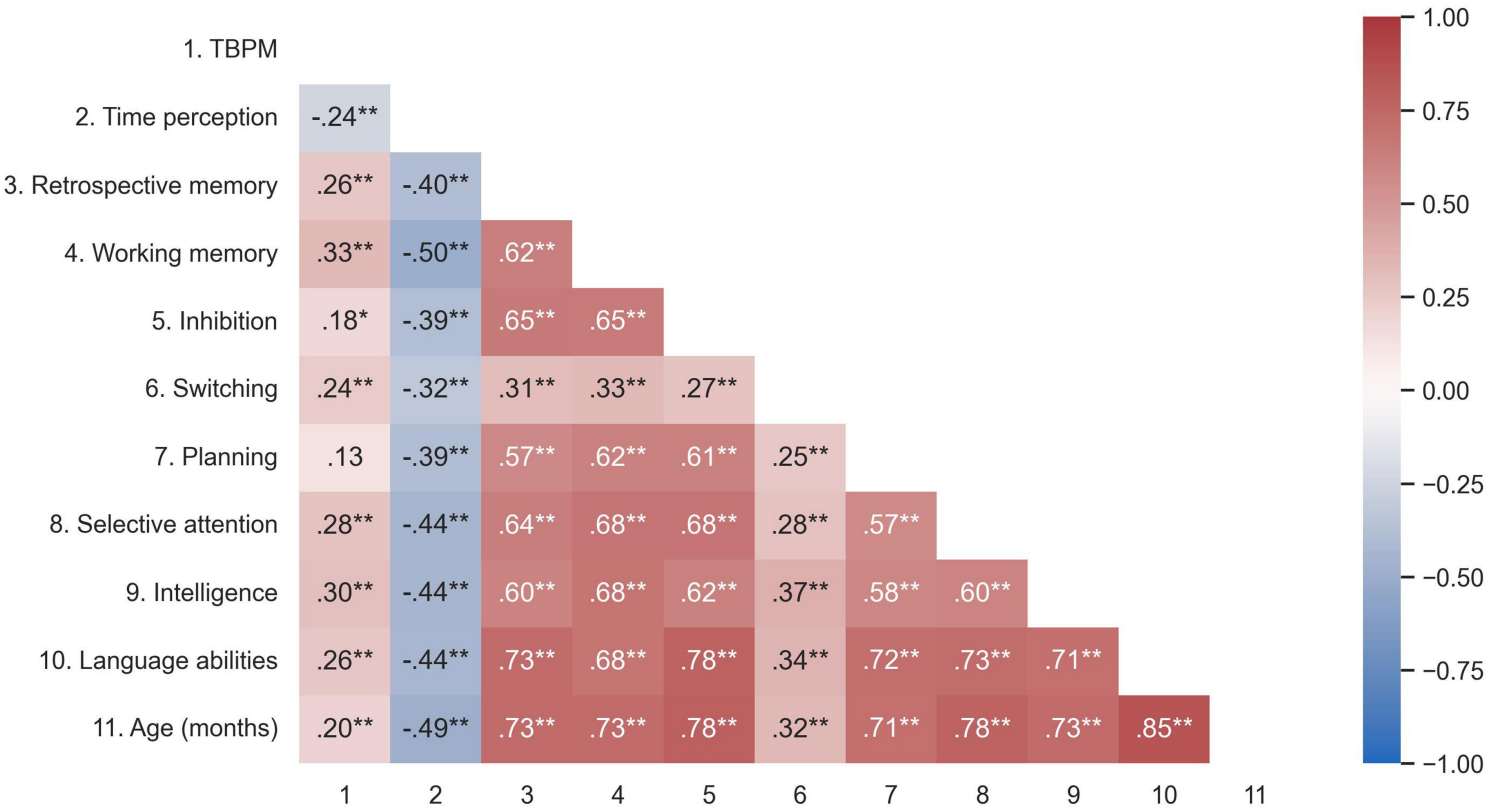
Pearson’s correlation heatmap of TBPM, age, cognitive measures, and time perception. * p < 0.05; ** p < 0.01.

Regarding the co-occurrence between TBPM and cognitive tasks, the study revealed a positive correlation with inhibitory control ( p < 0.05), WM ( p < 0.01), switching ( p < 0.01), intelligence ( p < 0.01), RM ( p < 0.01), attention ( p < 0.01), and language abilities ( p < 0.01). On the other hand, TBPM exhibited a negative correlation with time perception ( p < 0.01, as expected). No correlation was observed between TBPM and planning ( p > 0.05). The intercorrelations of cognitive measures have been presented in Figure 4.

Considering the age factor, among three-year-old’s, a negative correlation between TBPM and time perception was observed ( r = −0.32, p < 0.05). In the group of four-year-old’s, positive correlations were observed between TBPM and WM ( r = 0.40, p < 0.05), intelligence ( r = 0.35, p < 0.05), attention ( r = 0.46, p < 0.01), and languages abilities ( r = 0.42, p < 0.01). In the group of five-year-old’s, a positive correlation was observed between TBPM and attention ( r = 0.32, p < 0.05), as well as language abilities ( r =0.35, p < 0.05), while a negative correlation was found with time perception ( r = −0.34, p < 0.05). On the other hand, among the oldest children (six-year-olds), a positive correlation was observed between TBPM and WM ( r = 0.47, p < 0.01), switching ( r = 0.37, p < 0.05), and intelligence ( r = 0.33, p < 0.05). The complete correlation matrix of TBPM with cognitive abilities, divided into age groups, has been presented in Table 5.

**Table 5:** Correlation matrix of TBPM and cognitive measures between age groups.

TBPM	Age group				
	2	3	4	5	6
Time perception	0.01	−0.32*	0.01	−0.34*	−0.20
Retrospective memory	0.21	−0.24	0.31	0.18	0.28
Working memory	−0.20	0.11	0.40*	0.21	0.47**
Inhibitory	−0.01	−0.31	0.26	0.07	0.16
Switching	−0.27	0.16	−0.01	0.23	0.37*
Planning	−0.13	−0.26	−0.05	0.10	0.01
Attention	0.25	−0.13	0.46**	0.32*	0.10
Intelligence	0.27	0.10	0.35*	0.20	0.33*
Language abilities	0.10	−0.27	0.42**	0.35*	0.26

** *p* < 0.01; * *p* < 0.05.

### 3.4 Predictors of TBPM task performance

To identify the predictors of TBPM in the whole sample, a multiple regression procedure was employed. The initial model consisted of the following predictors: attention, planning, WM, switching, time perception, language abilities, RM, inhibitory control, age in months, frequency at which the participants glanced at the hourglass, and intelligence.

The regression results indicate that the predictors of TBPM are the frequency at which the participants looked at the hourglass (*B* = 0.32, *β* = 0.60, *t* = 9.42, *p* < 0.001) and WM (*B* = 0.05, *β* = 0.19, *t* = 2.01, *p* < 0.05). The model presented in Table 6 explains approximately 53% of the variance in TBPM scores ( $R^{2}$ =0.53, $F_{\left(11,144\right)}$ = 14.9, p < 0.001). Considering the age group division, only in the group of 2-year-olds, the number of glances at the hourglass (*B* = 0.12, *β* = 0.22, *t* = 0.24, *p* > 0.05) was not found to be a statistically significant predictor of TBPM. On the other hand, WM does not emerge as a statistically significant predictor across the age groups. The values of predictor coefficients with age group division have been included in Table 2A (see Appendix).

**Table 6:** Predictors’ coefficient of multiple regression.

Predictor	B	SE	*β*	t	p
Intercept	−0.64	0.99		−0.65	0.516
Number of glances at the hourglass	0.32	0.03	0.60	9.48	0.001
Age (in months)	0.01	0.05	0.08	0.27	0.790
Attention	<0.01	0.01	<0.01	0.01	0.990
Switching	0.03	0.07	0.03	0.40	0.689
Intelligence	0.05	0.04	0.12	1.39	0.168
Retrospective memory	0.06	0.05	0.10	1.27	0.208
Time perception	0.06	0.23	0.02	0.25	0.803
Inhibitory	−0.01	0.01	−0.06	−0.63	0.528
Working memory	0.05	0.02	0.19	2.01	0.047
Language abilities	0.02	0.01	0.19	1.64	0.103
Planning	−0.10	0.08	−0.10	−1.25	0.214

*R*^2^ = 0.53; *F*_(11,144)_ = 14.90; p < 0.001; $VIF_{\mathrm{all}}$ <5.

## 4 Discussion

As the majority of everyday PM challenges have, in fact, a time-based nature (see, e.g., Ellis and Nimmo-Smith, 1993), the current study yields important conclusions regarding essential aspects of children’s cognition. We addressed four research questions: 1. When can we observe the first signs of children’s ability to remember to initiate a delayed intention on their own after a specific time has elapsed?; 2. Are there age-related differences in TBPM performance among preschoolers?; 3. What role does time monitoring play in children’s TBPM performance?; 4. Are there cognitive predictors of TBPM performance in preschoolers?

The performance level of TBPM by 2-year-old children was greater than zero. This finding demonstrates that when the study’s methodology is adapted to the children’s abilities (e.g., using an hourglass instead of a watch, enhancing motivation through a game context), even children as young as 2-year-olds can perform TBPM tasks. However, their ability to remember to perform actions at specific times or after a delay is still limited (almost half if the child never turned the hourglass). In fact, the level of TBPM performance in all age groups was very low.

We anticipated that, according to the multi-process theory (McDaniel and Einstein, 2000) and the Executive Framework (Mahy et al., 2014), due to the cognitive resource-intensive nature of TBPM, involving continuous, effortful time monitoring, task performance would exhibit age-related improvements. We further predicted that age-related enhancements in PM would be contingent upon the maturation of specific EF. Surprisingly, the study’s findings did not align with our expectations. All children performed at a similarly low level, with close to half of the participants in each age group struggling to complete the task. This uniform distribution of results might indicate that within the analyzed cohort, these skills were still at an early developmental stage, and age-related differences might only manifest later in development (*cf.* Ceci and Bronfenbrenner, 1985; Aberle and Kliegel, 2010).

Examining TBPM in preschool-aged children poses a significant challenge due to difficulties in selecting an appropriate prospective task. Consequently, there is a possibility that the nature of the task may have influenced the results. Despite incorporating an hourglass and adhering to play conventions, our task proved to be very challenging for the majority of children. It would be worthwhile to examine the reliability of the task using the test–retest method in future research to ensure that the nature of the TBPM task was not the reason for the lack of findings regarding age differences and predictors of TBPM task performance.

PM appears to be intricately linked with context and environmental demands. Therefore, it can be hypothesized that even children with high cognitive capabilities might face challenges in the absence of exposure to the necessity of time monitoring. This perspective could shed light on the absence of age-related differences in TBPM, suggesting that the development of TBPM may be strongly influenced, for example, by natural environment and the demands imposed by it. When these demands are placed upon a child, numerous opportunities for the practice of PM tasks are created, thereby fostering the development of PM. Consequently, if a child is required to remember to execute certain tasks at a designated time in the future, this can be attributed to the specific dynamics of the household. In the context of subsequent research endeavors, it would be valuable to investigate the extent to which parents stimulate their children. Such an exploration may reveal age-related differences among those who receive higher levels of stimulation. Although our study lacked data on this aspect, it is advisable to incorporate it into future research, as it could emerge as a pivotal factor. This aligns with the findings from the study conducted by Zięba et al. (2021), which showed that children whose parents were excessively protective exhibited lower PM performance than those with less protective parents.

Additionally, the experimenter’s involvement in turning the hourglass after a missed opportunity, intended to ensure equal chances for each participant, may have introduced potential issues. This procedural aspect could function as a reminder, raising concerns about the self-initiation of retrieval. Moreover, the frequent reminders may alter the nature of the task, potentially shifting it toward a vigilance or short-term memory activity rather than maintaining its intended status as a PM task. On the other hand, not implementing this reminder-based approach could introduce a floor effect, where participants might struggle to meet the task requirements. To address these considerations and enhance the robustness of future studies, incorporating two conditions in future studies – one with the experimenter turning the hourglass and one without, would provide a more nuanced understanding of participant performance.

The utilization of the strategy involving a glance at the hourglass (i.e., time monitoring) has been shown to enhance task performance, as evidenced by findings among preschool children (Geurten et al., 2016) and even elderly adults (Mioni and Stablum, 2014; Mioni et al., 2020). This underscores the significance of time monitoring and corroborates the importance of time checking behavior among preschoolers. It is worth noting that despite the presence of this strategy, it remains relatively underdeveloped. In adults, a substantial difference exists between the number of glances in the first and last 15 s (Schmidt et al., 2023). Adults tend to check the time more frequently toward the end, a pattern similarly observed in 10–14-year-old children (Ceci and Bronfenbrenner, 1985). Such relationships were not evident within the examined group, and the correlation with TBPM task performance appeared at a moderate level.

The pattern of associations between cognitive abilities and TBPM varies across different age groups. More correlations become apparent in children from the age of 4, where WM, intelligence, attention, and language abilities play specific roles. In 6-year-olds, the relevant factors include WM, switching, and intelligence. Intriguingly, when constructing a comprehensive model using linear regression, age did not emerge as a significant predictor; instead, it was WM and time checking behavior. This suggests that cognitive abilities and proficient application of task monitoring strategies are pivotal regardless of age. This effect is particularly pertinent for practical implications, suggesting that teaching children strategies might contribute to enhancing their PM. However, the confirmation of this conclusion requires replication in subsequent studies. In future research on TBPM, it would be also worthwhile to delve deeper into the exploration of attention as a potential predictor, simultaneously serving as a mechanism influencing monitoring and, consequently, PM.

In summary, the study demonstrated that even 2-year-olds are capable of performing TBPM tasks. The next step should involve examining various factors beyond general cognitive development that hold significance in this context. It is crucial to emphasize that PM is genuinely essential in daily life for achieving independence and success, as seen in school or work settings. Consequently, exploring the mechanisms underlying its functioning through naturalistic studies, particularly in very young children, holds significant value.

### Conclusion

The results of the present study clearly indicate that even 2-year-olds can reliably succeed in TBPM tasks. From a conceptual perspective, our findings support time monitoring behavior as the underlying mechanism for the observed differences in TBPM performance.

## Data availability statement

The data analyzed in this study is subject to the following licenses/restrictions: the datasets used and analyzed during the current study are available from the corresponding author on reasonable request. Requests to access these datasets should be directed to ES, szpakiewicz.elzbieta@gmail.com.

## Ethics statement

The studies involving humans were approved by the Ethics Committee of the Jagiellonian University; approval number: KE/02/112017. The studies were conducted in accordance with the local legislation and institutional requirements. Written informed consent for participation in this study was provided by the participants’ legal guardians/next of kin.

## Author contributions

ES: Conceptualization, Data curation, Formal analysis, Funding acquisition, Investigation, Methodology, Project administration, Writing – original draft. NJ: Formal analysis, Writing – review & editing.
