# Supplementary material for: Time-based prospective memory in preschoolers – the role of time monitoring behavior
Source: Front Psychol. 2024 Feb 7;15:1276517. doi: 10.3389/fpsyg.2024.1276517 (PMC10879596; doi:10.3389/fpsyg.2024.1276517)
Supplement: Supplementary file 1 [file Table_1.pdf]

Table 1A. Frequencies of TBPM scores between age groups.

Age group	*TBPM score*	*Count*	%	*Cumulative* %
2	0	22	46.81	46.81
	1	12	25.53	72.34
	4	6	12.77	85.11
	2	6	12.77	97.87
	6	1	2.13	100.00
3	0	21	44.68	44.68
	1	12	25.53	7.21
	2	5	1.64	8.85
	3	4	8.51	89.36
	4	3	6.38	95.74
	6	1	2.13	97.87
	5	1	2.13	100.00
4	0	17	36.17	36.17
	1	9	19.15	55.32
	2	7	14.89	7.21
	4	6	12.77	82.98
	6	2	4.26	87.23
	3	2	4.26	91.49
	10	1	2.13	93.62
	9	1	2.13	95.74
	7	1	2.13	97.87
	5	1	2.13	100.00
5	0	20	40.00	40.00
	3	7	14.00	54.00
	2	7	14.00	68.00
	1	6	12.00	80.00
	9	2	4.00	84.00
	7	2	4.00	88.00

	6	2	4.00	92.00
	10	1	2.00	94.00
	8	1	2.00	96.00
	5	1	2.00	98.00
	4	1	2.00	100.00
6	0	23	46.94	46.94
	2	9	18.37	65.31
	6	4	8.16	73.47
	1	3	6.12	79.59
	9	2	4.08	83.67
	7	2	4.08	87.76
	5	2	4.08	91.84
	4	2	4.08	95.92
	3	2	4.08	100.00
