# Supplementary material for: Time-based prospective memory in preschoolers – the role of time monitoring behavior
Source: Front Psychol. 2024 Feb 7;15:1276517. doi: 10.3389/fpsyg.2024.1276517 (PMC10879596; doi:10.3389/fpsyg.2024.1276517)
Supplement: Supplementary file 2 [file Table_2.pdf]

Table 2A. TBPM predictor's coefficients in different age groups.

Age group	Predictor	*B*	*SE*	β	*t*	*p*
2	Intercept	-1.74	5.52		-0.31	.769
	Time perception	0.31	0.71	.20	0.43	.690
	Number of glances at the hourglass	0.12	0.51	.22	0.24	.822
	Retrospective memory	-0.05	0.32	-.08	-0.17	.871
	Working memory	-0.10	0.18	-.33	-0.58	.596
	Inhibition	<0.01	0.06	-.01	-0.01	.995
	Switching	-0.13	0.57	-.13	-0.24	.825
	Planning	-0.01	0.71	-.09	-0.16	.882
	Selective attention	0.05	0.09	.42	0.62	.567
	Inteligence	0.25	0.24	.56	1.04	.357
	Language abilities	<0.01	0.11	-.04	-0.03	.975
3	Intercept	-2.01	2.94		-0.68	.508
	Time perception	0.08	0.49	.04	0.17	.868
	Number of glances at the hourglass	0.32	0.11	.74	2.96	.012
	Retrospective memory	0.01	0.11	.01	0.05	.958
	Working memory	-0.02	0.09	-.05	-0.21	.836
	Inhibition	-0.01	0.02	-.11	-0.48	.640
	Switching	0.35	0.26	.25	1.35	.203
	Planning	-0.11	0.26	-.10	-0.43	.675
	Selective attention	-0.02	0.04	-.11	-0.49	.631
	Inteligence	0.07	0.08	.18	0.97	.353
	Language abilities	0.01	0.03	.07	0.29	.774
4	Intercept	-2.78	3.23		-0.86	.400
	Time perception	0.44	0.56	.13	0.78	.444

	Number of glances at the hourglass	0.23	0.07	.57	3.35	.003
	Retrospective memory	0.05	0.12	.07	0.43	.674
	Working memory	0.06	0.08	.13	0.70	.495
	Inhibition	0.03	0.03	.13	0.89	.384
	Switching	-0.28	0.19	-.24	-1.49	.153
	Planning	-0.22	0.18	-.19	-1.21	.240
	Selective attention	0.01	0.04	.04	0.28	.783
	Inteligence	0.10	0.08	.17	1.14	.268
	Language abilities	0.03	0.03	.21	0.95	.354
5	Intercept	-5.56	3.60		-1.55	.132
	Time perception	-0.60	0.61	-.15	-0.99	.332
	Number of glances at the hourglass	0.34	0.10	.50	3.28	.002
	Retrospective memory	0.10	0.12	.12	0.83	.412
	Working memory	<0.01	0.06	-.01	-0.07	.941
	Inhibition	<0.01	0.04	-.01	-0.07	.945
	Switching	0.05	0.19	.04	0.26	.800
	Planning	-0.05	0.19	-.04	-0.28	.778
	Selective attention	<0.01	0.04	<.01	-0.03	.978
	Inteligence	0.09	0.09	.16	1.05	.302
	Language abilities	0.05	0.05	.18	1.08	.289
6	Intercept	-0.61	2.84		-0.22	.830
	Time perception	-0.10	0.56	-.02	-0.18	.860
	Number of glances at the hourglass	0.40	0.06	.73	7.10	<.001
	Retrospective memory	<0.01	0.10	<.01	0.01	.995
	Working memory	0.04	0.04	.16	1.15	.256
	Inhibition	-0.01	0.03	-.05	-0.39	.696
	Switching	0.14	0.14	.12	1.04	.306
	Planning	-0.19	0.14	-.13	-1.32	.195
	Selective attention	-0.01	0.03	-.06	-0.52	.605

	Inteligence	<0.01	0.06	.01	0.05	.960
	Language abilities	0.02	0.04	.06	0.54	.595
